# Evaluation of the effectiveness of student learning and teacher instruction on team-based learning during quality control of diagnostic imaging

**DOI:** 10.1080/10872981.2020.1732159

**Published:** 2020-02-23

**Authors:** Meng-Fang Tsai, Jo-Chi Jao

**Affiliations:** a General Research Service Center, National Pingtung University of Science and Technology, Neipu, Pingtung, Taiwan; b Department of Medical Imaging and Radiological Sciences, Kaohsiung Medical University, Kaohsiung, Taiwan

**Keywords:** Team-based learning, action research, instructional development, student learning, radiologic technology, reflective practice

## Abstract

**Background**: Team-Based Learning (TBL), which is a student-centered instructional approach, has been applied in various health-related courses, but research on the effectiveness of TBL in radiologic technology is limited. More research is needed to examine the effectiveness of TBL within the field of radiologic technology as well as to study teachers’ reflective practices for instructional development in TBL.

**Objectives**: This study examines the effectiveness of TBL on students’ learning and course instructors’ instructional development during quality control activities in diagnostic imaging.

**Design**: This study employed an action research approach with mixed-methods. The study was categorized using four TBL modules as the topics: film/screen receptors and processors, radiography, mammography, and computed tomography. Quantitative data included pre-test scores on individual readiness assurance tests (IRAT-pre), group readiness assurance tests (GRAT), and post-test scores on individual readiness assurance tests (IRAT-post). Qualitative data included students’ responses to open-ended questions about their experience with TBL and transcripts of instructors’ interviews.

**Results**: Forty junior college students participated in the study. A non-parametric test was conducted to compare the scores. The results showed that the GRAT score was significantly higher than the IRAT-pre-score, and the IRAT-post score was significantly higher than the IRAT-pre-score. The IRAT-post score was significantly higher than the GRAT score for the first and fourth modules, but IRAT-post score was significantly lower than the GRAT score on the second and third modules. Using direct content analysis, five themes were coded around instructional development, while 15 themes were coded to understand students’ experiences with TBL.

**Conclusions**: TBL can be an effective instructional approach to improve students’ understanding of radiologic technology content. The results of this research can help instructors decide what action plan to implement to increase the effectiveness of TBL when further employing it for radiologic technology courses.

## Introduction

It has become essential for instructors to develop college students’ collaborative skills [1,2]. Lecture-based instruction is currently the most commonly used teaching strategy. The lecture format works because teachers are typically the primary transmitters of knowledge, while students are the knowledge receivers. However, although lecture-based instruction can be effective, it tends to create less engagement in learning [3–5]. Obtaining high-quality radiologic images for accurate diagnosis is the main responsibility of radiologic technologists; therefore, it is crucial to create a learning environment that allows students to develop their collaborative and higher-order learning skills, as they can then better develop their knowledge and application skills for how to obtain high-quality images and correct diagnosis through completing classroom tasks in teams. In this study, we found that the Radiology Technology instructor (the corresponding author of this study) experienced her students playing on their cellphones while attending lectures. Students were easily bored and did not pay attention or were simply absent from class. Therefore, the instructor decided to change her traditional teaching method of lecture-based instruction to Team-Based Learning (TBL). She instructed her students to come to class prepared for interactive collaboration and content knowledge acquisition using group work. The goal of this study was to understand the effectiveness of student learning and instructor levels of development in teaching by employing an action-research methodology with a mixed-method approach.

In large classes, teachers face a major challenge in creating opportunities for students to interact collaboratively. TBL can provide an effective instructional approach that gives students the chance to learn alongside their peers in an interactive way. A TBL module has seven components: 1) pre-class preparation; 2) an individual readiness assurance test (IRAT); 3) a group readiness assurance test (GRAT); 4) an appeal; 5) a mini-lecture; 6) applications; and 7) peer evaluation [6,7]. TBL is designed to use teams as the primary format for conducting group discussion activities during class time. Students depend on each other to complete activities within each group, and they are accountable as a group to complete group tasks [8–12]. TBL instructors create a learning climate with mutual respect in which students take responsibility for their own learning [13–17]. It is a unique method in that it encourages students to build their understanding and knowledge through a social format of group discussion.

The research results indicate that students improved their understanding of content in TBL classes and that lower-achieving students benefited the most from this particular teaching approach [18–21]. Students reported positive feedback and performed better in groups than as individuals in TBL classes [22]. To enhance students’ motivation, enjoyment, engagement, cooperative skills, and higher-level learning such as problem-solving skills and critical thinking, TBL is an effective teaching approach to use for large classes [7,23–29].

Additionally, research has shown that teachers’ instructional development can be greatly influenced by their reflections on their teaching practices [30,31]. Reflection and reflective practice can play an important role in helping develop professional competence in health professions [2,30–33]. Reflecting on their teaching can help teachers gain an understanding of what teaching areas they need to improve to enhance students’ learning. It is vital for teachers to progressively develop their instructional competence, and reflection is an effective way to accomplish this. This study adopted an interview approach to examine the instructor’s development during her use of TBL over one semester.

The effectiveness of TBL has been found to enhance students’ academic performance in health science fields such as nursing, anatomy, pediatrics, pharmacy, medical ethics, physiology, neurology, and psychology [14,15,17,22,29,34–46]. However, little research exists that examines the effectiveness of students’ learning and instructors’ development in TBL in the field of radiologic technology.

## Research questions

This study aimed to investigate students’ learning effectiveness from a TBL course and an instructor’s pedagogical development of TBL over one whole semester in 2015. Three research questions are proposed:

1. Did students improve their performance in TBL classes?
2. How did students perceive TBL classes and their learning in TBL classes?
3. How did the instructor develop her TBL teaching over the semester?

## Materials and methods

### Research design

Action research with a mixed-method approach was employed in this study. Action research involves using course teachers as researchers who follow a four-step process of conducting research [47]. The four steps are: 1) identify an area of focus; 2) collect data, 3) analyze and interpret the data; and 4) develop an action plan. This research type follows a systematic inquiry for information collection of course instructors’ insightful and reflective practices with the goal of making positive changes to further improve students’ learning. Furthermore, because it is vital for course instructors to collect sufficient data sources to achieve the purposes of the study, a mixed-method approach was employed [48]. Action research follows reciprocal processes in a model: planning for action, action for change, and results for further planning. Exemption was approved by the Institutional Review Board (IRB) of Kaohsiung Medical University Hospital, Taiwan.

### Context and participants

This study was conducted during the course *Quality Control of Diagnostic Imaging*, which was offered to junior students studying in the Department of Medical Imaging and Radiological Sciences during the spring semester of 2015 in a medical university. The course was an 18-week 2-credit required course. Forty junior college students participated in the study.

### Instruction design and procedures

In the first class, the course instructor communicated with students about their responsibility to learn in the course as well as in TBL modules. Students were also informed about which classes and topics would be taught using TBL and how the scores from the TBL modules would be calculated into their final course grades. Four TBL modules were designed and scheduled, each with a different topic: (1) Film/screen receptors and processors; (2) Radiography; (3) Mammography; and (4) Computed tomography. Table 1 provides information about the number of weeks and the specific topic in each TBL class.

**Table 1.** Schedule and topics of four TBL modules

Week	Topic
7	The quality control of film/screen receptors and processors
10	The quality control of radiography
12	The quality control of mammography
14	The quality control of computed tomography

Students were grouped into teams of three or four members, with a total of 11 groups. Grouping was based on the order of the student list. On the list, Nos. 1 to 4 are the first group, Nos. 5 to 9 are the second group, and so on. The last group had only three members. In order to have students sitting closer for performing calculations together and having more opportunities for communication, each group was designed to have only 3–4 members; instead of grouping more than seven team members in a typical TBL. The TBL teams remained the same for the entire course. In the course, students were expected to learn the principles of diagnostic imaging and how to perform quality control for diagnostic imaging; furthermore, they needed to show their competence in obtaining high-quality images. Each TBL module consisted of pre-class preparation, multiple choice questions in an IRAT, a GRAT, an appeal, in-class application activities, a mini-lecture, and peer evaluation. There were two IRAT timelines in each TBL module: IRAT-pre and IRAT-post. IRAT-pre was performed at the beginning of each module and IRAT-post was performed at the end of each module. Each IRAT-pre and GRAT had the same ten test items except Module 3. GRAT in Module 3 had five more items than the IRAT-pre for teams to have more exercises. Scratch cards, a kind of immediate feedback assessment technique (IF-AT), were used during GRAT. The application activity contained 4–5 multiple choice questions using four Ss structures: a significant problem, the same problem, a specific choice, and simultaneous reporting. IRAT-post, a modification of typical TBL, was designed to encourage students’ participations in team discussions and to know whether students’ learning outcomes differed between IRAT, GRAT and IRAT-post after team discussions. IRAT-post had 14–15 test items that were slightly modified from the GRAT and activity questions with the same stem, but with different order or contents of options of multiple choice questions. Only the same or similar ten questions in IRAT-pre, GRAT and IRAT-post were used for the evaluation of the effectiveness of student learning. After the IRAT-post, peer evaluation was performed. Students gave a weighting number to their team members according to their contribution to the team. The sum of the weighting number to team members was equal to the number of group members minus one. The duration time of each in-class activity is shown in Table 2.

**Table 2.** Time duration of TBL procedures for each module

In-class activities	Time duration (min)
IRAT-pre	15
GRAT	20
Appeal	10
Mini-lecture	10
Application	20
Feedback and peer evaluation	10
IRAT-post	15
Total	100

Each TBL counts as 5% of the final academic score. The grading rule for each TBL is shown in Table 3. The peer-review was evaluated according to the feedback given by other group members within the same group. The pre-reading materials were posted on the e-learning platform in advance. Students were required to finish the pre-readings before attending TBL classes.

**Table 3.** Item and percentage of TBL grading

item	percentage
IRAT-pre	35%
GRAT	15% (peer-review weighted)
Application	15% (peer-review weighted)
IRAT-post	35%

### Data collection

Quantitative data included IRAT-pre-scores, GRAT, and IRAT-post scores collected in each TBL class. Qualitative data contained students’ responses to six survey questions at the end of the semester and the teacher’s interview to gather her reflections on her TBL experience. Descriptions of the six survey questions and the teacher interview are provided in Table 4.

**Table 4.** Description of open-ended questions and teacher interview questions for reflection

Question	Description
1	How much time did you spend on pre-class preparation?
2	Do you consider the amount of pre-class preparation materials suitable for you? Why?
3	Do you consider the difficulty level of pre-class preparation content suitable for you? Why?
4	In the TBL class, most in-class activities were conducted through group discussion. Did this method influence your learning? How did it influence your learning and why?
5	Do you hope the course instructor continues to use TBL in future courses? Why?
6	Do you have any suggestions for the TBL classes this semester?
	Description for teacher interview
1	First of all, I would like to ask what the topic of the TBL class was last time.
2	How did you design the TBL class last time? Why?
3	Did the instructional procedures you designed go well as expected in the class? Why?
4	Was students’ learning in TBL class as expected? Why?
5	After teaching the TBL class, would you modify the way to assign pre-class preparation and test design materials, or the methods of running appeals, mini-lectures, or applications? Why?

### Data analysis

This study employed a repeated measure design to compare IRAT-pre, GRAT, and IRAT-post data collected in each module. A nonparametric Wilcoxon sign rank test was used to examine students’ understanding of diagnostic imaging quality control by comparing the IRAT-pre-scores with GRAT, IRAT-pre-scores with IRAT-post scores, and GRAT with IRAT-post scores.

Directed content analysis was used to code students’ responses to four open-ended questions, indicating students’ learning experience in TBL; it was also used to code transcriptions of teacher interviews demonstrating their instructional development over the semester [49]. This analysis method codes the themes emerging from the data. The frequencies and themes coded from the data are presented in the results section.

Coding qualitative data contained two parts. As students’ reports to survey questions 1–3 and part of students’ reports to survey questions 4–5 were numbers, suitable vs. unsuitable, yes vs. no, ambiguity (no indication to yes or no), or no response, we used frequencies to show the coding results of this part. Since students’ reports to survey question 6 and part of students’ reports to survey questions 4–5 were descriptions, we analyzed descriptive data based on semantic meaning that could relate to TBL learning, without counting the times on the occurrences of exact words in the text. A theme was given to each identified description based on its semantic meaning. The first author of the study first coded the qualitative data, including students’ qualitative responses and transcriptions of the instructor’s interviews. By coding the transcriptions of the instructor’s interviews, five themes were coded and identified. By coding students’ qualitative responses to three open-ended questions, fifteen themes were coded and identified. The fifteen themes were further grouped into four major categories that indicated broader meanings for the fifteen themes. The corresponding author who was the instructor of the course further checked and confirmed the coding analysis and results.

## Results

### Quantitative results

We conducted statistical analysis on 40 students’ scores. Table 5 shows the mean and standard deviation of the IRAT-pre, GRAT, and IRAT-post. Analytical results of the nonparametric Wilcoxon sign rank test show that the mean score on the GRAT was significantly higher than that of the IRAT-pre in all four modules. Except for in Module 3, the mean score on the IRAT-post was significantly higher than on the IRAT-pre. The mean score of IRAT-post in Modules 1 and 4 was significantly higher than on the GRAT; however, the mean score of the IRAT-post in Modules 2 and 3 was significantly lower than the mean score on the GRAT.

**Table 5.** Comparison of TBL scores on IRAT-pre, GRAT, and IRAT-post scores

Module	IRAT-pre-Score	GRAT Score	IRAT-post Score	P value^a^	P value^b^	P value^c^
1	51 ± 21	87 ± 10	94 ± 12	P < 0.001	P < 0.001	P < 0.01
2	51 ± 19	85 ± 10	78 ± 15	P < 0.001	P < 0.001	P < 0.01
3	61 ± 20	91 ± 10	64 ± 14	P = 0.250	P < 0.001	P < 0.001
4	52 ± 16	80 ± 11	97 ± 6	P < 0.001	P < 0.001	P < 0.001
All	54 ± 19	86 ± 11	83 ± 18	P < 0.001	P < 0.001	P = 0.192

^a^indicates IRAT-pre vs. IRAT-post.

^b^indicates IRAT-pre vs. GRAT.

^c^indicates GRAT vs. IRAT-post.

### Qualitative results

#### Results of students’ responses

We used frequencies to demonstrate the analytical results of Questions 1–5 and to present the themes coded from students’ qualitative responses for Questions 4–6. Analytical results were presented as follows based on each question.

Q1. How much time did you spend on pre-class preparation?

According to students’ responses, fifty percent of students spent less than three hours doing pre-class preparation. However, a few students spent more than 6 hours in pre-class preparation. No students reported they did not spend any time on the pre-class preparation. The hours students spent on pre-class preparation were grouped into different time segments. The frequency of the different time segments is presented in Table 6.

**Table 6.** Frequency of hours students spent on pre-class preparation

Hours spent	Frequency
0–3 hours	20 students
3–6 hours	12 students
>6 hours	5 students
No response	3 students
Total	40

Q2. Do you consider the amount of pre-class preparation materials suitable for you? Why?

Most students felt that the amount of pre-class preparation assigned by the instructor was suitable. Several students reported the amount as unsuitable and ambiguous. “Ambiguous” indicates that students’ responses did not directly indicate a yes or no. The frequency of answers on the suitability of pre-class preparation materials is shown in Table 7.

**Table 7.** Coding of frequency of students’ responses to four open-ended questions

	Frequency of response coded			
Questions	Yes	No	Ambiguous	No response
2. Do you consider the amount of pre-class preparation materials suitable for you?	26	6	5	3
3. Do you consider the difficulty level of pre-class preparation content suitable for you?	18	13	5	4
4. In the TBL class, most activities are run in group discussions. Does group discussion influence your learning?	35	2	0	3
5. Do you hope that the course instructor continues to use TBL in the course in the future?	35	2	0	3

Here, we present three students’ responses indicating why they felt the amount unsuitable:

S12: … because it is related to pre-class preparation and IRAT, I want to fully understand the content of PPT, maybe understanding one PPT slide has already cost me a lot of time

S15: … English materials are difficult

S25: because new content needs to spend more time preparing. If the amount is too much, the preparation is unfinished.

Here, we present three students’ responses to show why they felt the pre-class preparation amount was ambiguous:

S22: If the material was about principles, the amount was suitable. If the material was about quality control, the amount was too much.

S26: If the material was purely about image, principles, the amount was suitable and could help us to review the content learned … .if the material of quality control was added, the content was complex and abstract that increased students’ frustration from doing pre-class preparation.

S31: sometimes the material amount was suitable; sometimes the material amount was unsuitable; there are keywords hard to understand in PPT.

Q3. Do you consider the difficulty level of pre-class preparation content suitable for you? Why?

Approximately 45 percent of students felt that the difficulty level of the pre-class preparation content assigned by the instructor was suitable. Approximately 32.5 percent of students reported the difficulty level unsuitable. The frequency of answers on suitability of difficulty level of pre-class preparation materials is shown in Table 7.

Q4. In the TBL class, most activities are run in group discussions. Does group discussion influence your learning? Through which way and why?

Eighty-eight percent of students reported that group discussion influenced their learning. Nine themes were coded on the second level: 1) multiple types of test items; 2) pre-class preparation; 3) peer evaluation; 4) team effectiveness; 5) content understanding; 6) learning strategy; 7) fun; 8) concentration; and 9) learning interest.

Q5. Do you hope that the course instructor continues using TBL in the course in the future? Why?

Eighty-eight percent of students indicated that TBL should continue to be used in the course. Ten themes were coded on the second level for why students thought TBL should continue to be used: 1) multiple types of test items; 2) pre-class preparation; 3) mini-lecture; 4) team effectiveness; 5) content understanding; 6) learning strategy; 7) fun; 8) concentration; 9) learning interest; and 10) participation.

Q6. Do you have any suggestions for the TBL classes this semester?

There were 47.5% students who provided suggestions for the TBL class. Eight themes were coded on the second level according to the suggestions: 1) multiple types of test items; 2) pre-class preparation; 3) semester design; 4) time management; 5) mini-lecture; 6) teaching strategy; 7) team formation; and 8) content understanding.

The themes coded from the responses to three open-ended questions were grouped into two levels and are compiled in Table 8, with students’ excerpts from their responses provided as coding examples along with each theme in the table.

**Table 8.** Themes coded from the responses to three open-ended questions and excerpts provided

First Level	Second Level	Excerpt	Q4	Q5	Q6
TBL Instructional Elements	Multiple types of test items	S12: My understanding is strengthened. I can also understand other’s opinions. The test items show diversification and it is wonderful to not limit the content within PPT. (Q4)	✓	✓	✓
	Pre-class preparation	S8: I sometime don’t understand the content of PPT in pre-class preparation materials. (Q6)	✓	✓	✓
	Peer evaluation	S15: To do peer evaluation is to contribute to each other’s learning. (Q4)	✓		
	Semester design	S12: 1. I hope the whole TBL lesson can change to 1hr TBL+1hr lecture on PPT.; 2. The materials for pre-class preparation could be less so I could be better fully prepared.; 3. The teacher could have more time on lecturing with PPT.; 4. I feel it would be better if we could have 1hr TBL and 1hr lecture every week and cover less content. (Q6)			✓
	Time management	S8: Time management of class time should better improve. (Q6)			✓
	Mini-lecture	S28: I had better impressions when I read before class and then I had better understandings when the teacher lectured on the content in the class (Q5)		✓	✓
	Teaching strategy	S33: I hope we could use IRS to give the answers so we wouldn’t always fail giving the answers with not raising the cards in time. (Q6)			✓
Team	Team effectiveness	S1: TBL can strengthen my impressions, and it is more interesting to learn in groups than doing pre-class preparation by myself. (Q5)	✓	✓	
	Team formation	S19: We can have different group members in every class. In some groups, there are members actually only listening to others’ opinions. (Q6)			✓
Learning	Content understanding	S12: My understanding is strengthened. I can also understand other’s opinions. The test items show diversification and it is wonderful to not limit the content within PPT. (Q4)	✓	✓	✓
	Learning strategy (brainstorming, imagination)	S16: In the process of group discussions, I could know the differences of learning strategies used between my peers and I. (Q4)	✓	✓	
Motivation	Fun	S15: TBL is fun and interesting. (Q5)	✓	✓	
	Concentration	S26: TBL is interesting. I can enhance students’ concentration and familiarity of test items. (Q5)	✓	✓	
	Learning interest	S37: Because I didn’t learn with this kind of learning experience before, I felt very innovative and easily got interests in learning. (Q4)	✓	✓	
	Participation	S19: At first I didn’t get used to this method and got bad feelings, but afterwards I felt this method actually could enhance my willingness and participation in learning. (Q5)		✓	

We checked the themes appearing in students’ responses. Q4 is about students’ reasons why group discussions influence their learning. Q5 is about students’ perceptions of the teacher continuing to use TBL. Q6 is about suggestions for TBL classes.

#### Results of teacher interviews

Transcriptions of the instructor’s three interviews were coded, and the resulting themes are shown in Table 9, with the instructor’s excerpts from her interviews provided as coding examples along with each theme in the table.

**Table 9.** Themes coded from the instructor’s three interviews and excerpts provided

Theme	Excerpt	1^st^ interview	2^nd^ interview	3^rd^ interview
Topic of course content	I feel I may modify the topics after my first TBL lesson. At the beginning, I chose the latter three topics related to three different instruments. After first TBL lesson, I would chose the latter three topics based on the instruments with simpler principles. (1^st^ interview)	✓	✓	✓
Difficulty level of pre-reading	I would let students focus on learning principles in pre-reading and discussion. Because testing needs real instruments, … it is hard to image what instruments look like when students read by themselves. So I modified the content of pre-reading. (2^nd^ interview)	✓	✓	✓
Time management of TBL teaching	There is one area to work on-that is time management of discussion activities. I was over time in the last TBL lesson. I paid more attention to time management of discussions this time. (3^rd^ interview)	✓	✓	✓
Test design	I had 5 more test items on GRAT and five application items. So maybe I don’t need the 5 more test items on GRAT as I need to consider time since I need to do post individual test (1^st^ interview)	✓	✓	✓
Use of multiple teaching strategies	We could have different actions each time when choosing groups to answer the questions. For example, I could see which group stands up the fastest, they could first answered the question. (3^rd^ interview)		✓	✓

## Discussion

This study examines students’ learning effectiveness in four TBL classes on topics of quality control during diagnostic imaging. The topics include: Film/screen receptors and processors (Module 1), Radiography (Module 2), Mammography (Module 3), and Computed tomography (Module 4). The results indicate that students performed better after discussing their understanding with their group members and show that students had positive learning experiences toward the end of the semester.

Students’ GRAT scores were significantly higher than their IRAT-pre-scores in the four modules. These findings mean that students performed better after they had a chance to discuss information with their peers in teams; this resulted in desired outcomes for students’ learning in TBL classes. When comparing IRAT-pre and IRAT-post scores, only Module 3 did not show significant differences. In module 3, IRAT-post had ten similar test items with IRAT-pre, which has the same stem but different contents of options of multiple choice questions. This might be the reason why the score of the IRAT-post in module 3 did not increase relative to the IRAT-pre. When comparing GRAT and IRAT-post scores, in Modules 2 and 3, IRAT-post scores were significantly lower than GRAT scores. These findings illustrate that students’ knowledge acquired through discussion may not guarantee further knowledge retention, which indicates different results from McInerney and Fink’s study [50]. It is possible that some teams did not carry on in-depth discussion; rather, they may have just voted on which answer they agreed upon for test item questions. Students who did not hold discussions or provide explanations for their mutual decisions on test items may have demonstrated lower IRAT-post scores. Overall, there was a positive correlation between IRAT scores and final examination scores (p < 0.01).

Fifty percent of students reported spending between zero and three hours on their *general time of doing pre-class preparation*, and stated that the *amount of pre-class preparation materials* was suitable. From the point of view of the instructor, 2–4 hours preparation would be appropriate. Forty percent of students perceived the *difficulty level of pre-class preparation materials* as suitable; however, 32.5% of students still perceived the difficulty level as unsuitable. These findings may indicate that students have different levels of prior knowledge about the content being learned. When students were asked whether they hope the instructor will continue using TBL in future courses, 88 percent said ‘Yes,’ indicating a majority of positive attitudes toward learning in the TBL class.

Four major themes were identified by coding students’ responses to the three open-ended questions. Students stated that the essential components (i.e. multiple types of test items, pre-class preparation, peer evaluation, semester design, time management, mini-lecture, and teaching strategy) that were related to the theme *Relation in TBL Instructional Elements* promoted their learning [51]. Students suggested that the use of the IF-AT card was fun, but that the instructor might consider using the interactive response system (IRS) to reduce the incidence of students peeking at other teams’ answers. During the activity, the instructor encouraged students to express themselves. The group raising their colored answer cards the fastest could get the opportunity to present their idea and get some bonus points. However, it was hard to determine which team was the fastest by visual assessment. IRS might avoid this confusion.

Students reported that the use of a team allowed them to make more of an effort and contribute more to the teamwork to deepen knowledge, correct misunderstandings about content knowledge, and review prior knowledge through group interaction and explanation [52,53]. TBL helped students increase their construction of content understanding and the use of brainstorming and imagination in group discussions; they reported developing habits of preview and review through pre-class preparation, and they are interested in their peers’ learning strategies [10]. As for motivation, TBL helped increase students’ concentration, interest, and participation in learning, and they had fun, which is consistent with students’ findings that they were motivated to participate in TBL [41,54].

Regarding overall learning experiences in TBL, most students emphasized the effectiveness of having a team for constructing knowledge through group discussions and to explore a variety of peer learning strategies [10]. Several students felt that using the IF-AT card was cool and fun. In addition, students reported that RATs played an effective role in helping them realize whether their preparation was sufficient and whether they understood the content: IRAT for their preparedness before class and GRAT for enhancing in-depth understanding through group discussion [41]. The instructor’s mini-lecture helped students deepen their content knowledge and gave detailed explanations for typical misconceptions. Students did not find the use of peer evaluation useful because most students made nearly equal contributions to their teams; therefore, in most situations, every student received the same score. Pre-class preparation helped students develop the habit of reviewing their work before class to reduce the pressure of preparing for mid-term and final exams.

These findings demonstrate that through group discussion, appeals, and mini-lectures, students can increase their understanding of content in TBL lessons because they have the opportunity to reveal misconceptions and correct learned content [10,52,53]. The themes coded from students’ excerpted comments are consistent with the core components of development in TBL classes. These findings can help a course instructor decide how to better design TBL courses to help students learn effectively and efficiently.

In the three instructor interviews, we learned that the instructor found it challenging to select appropriate topics and difficulty levels for the pre-reading. The instructor also found time management for the class difficult based on students having to take the IRAT-post toward the end of each module. The instructor also considered it challenging to appropriately allocate class time to each TBL component; decisions had to be made taking into account the number of test items to include in the GRAT and the application and how to weigh class time appropriately for the mini-lecture and group discussion. During the second interview, the instructor stated that she made several changes in designing the third TBL module. The instructor chose the topic *imaging principles* instead of *imaging tools* so that students could more easily understand the content based on the content of imaging tools being more abstract through only reading. The instructor also modified the number of test items from 15 to 10 on the GRAT and 5 to 4 on the application. Beginning with the third TBL module, the instructor began using multiple instructional strategies to motivate students to freely share their thoughts during class discussions.

After the instructor accumulated more TBL teaching experience, more themes were coded from the interviews. The findings could explain that the instructor’s skills could progressively develop as she made more reflections on her TBL teaching [55,56]. When teachers become active agents in finding ways to improve their teaching strategies, their cognition of needs for change can be stimulated, increasing their motivation to improve their instruction.

Interestingly, the themes coded from the instructor’s interviews correspond with the themes coded from students’ qualitative responses in several areas such as teaching strategy, time management, and test design. The findings demonstrate that the improvements the instructor perceived in TBL teaching match what the students perceived as crucial to them in TBL learning.

## Conclusion

Students’ understanding of quality control during diagnostic imaging was retained at the end of the lesson in the study. Therefore, we conclude that TBL can be an effective instructional design to improve students’ learning in the field of radiological technology. When teachers reflect on their teaching, they can improve their instructional development. Educators who would like to use innovative instructional strategies can reference the TBL lesson designed in this study to increase students’ motivation in learning as well as to enhance their understanding of the lesson content.
